# Biocompatibility of Biomaterials for Nanoencapsulation: Current Approaches

**DOI:** 10.3390/nano10091649

**Published:** 2020-08-22

**Authors:** Bwalya A. Witika, Pedzisai A. Makoni, Scott K. Matafwali, Billy Chabalenge, Chiluba Mwila, Aubrey C. Kalungia, Christian I. Nkanga, Alain M. Bapolisi, Roderick B. Walker

**Affiliations:** 1 Division of Pharmaceutics, Faculty of Pharmacy, Rhodes University, Makhanda 6140, South Africa; bwawitss@gmail.com (B.A.W.); p.makoni@ru.ac.za (P.A.M.); 2 Department of Basic Sciences, School of Medicine, Copperbelt University, Ndola 10101, Zambia; scott.matafwali@cbu.ac.zm; 3 Department of Market Authorization, Zambia Medicines Regulatory Authority, Lusaka 10101, Zambia; bchabalenge@zamra.co.zm; 4 Department of Pharmacy, School of Health Sciences, University of Zambia, Lusaka 10101, Zambia; chiluba.mwila@unza.zm (C.M.); ckalungia@unza.zm (A.C.K.); 5 Department of Medicinal Chemistry and Pharmacognosy, Faculty of Pharmaceutical Sciences, University of Kinshasa, P.O. Box 212, Kinshasa XI, Democratic Republic of the Congo; christian.nkanga@unikin.ac.cd; 6 Department of Chemistry, Faculty of Science, Rhodes University, Makhanda 6140, South Africa; g18b2522@campus.ru.ac.za

**Keywords:** biocompatibility, haemocompatibility, histocompatibility, cytotoxicity, genotoxicity, nanospheres, liposomes, micelles, nanocrystals, nanoencapsulation, polymers, surfactants

## Abstract

Nanoencapsulation is an approach to circumvent shortcomings such as reduced bioavailability, undesirable side effects, frequent dosing and unpleasant organoleptic properties of conventional drug delivery systems. The process of nanoencapsulation involves the use of biomaterials such as surfactants and/or polymers, often in combination with charge inducers and/or ligands for targeting. The biomaterials selected for nanoencapsulation processes must be as biocompatible as possible. The type(s) of biomaterials used for different nanoencapsulation approaches are highlighted and their use and applicability with regard to haemo- and, histocompatibility, cytotoxicity, genotoxicity and carcinogenesis are discussed.

## 1. Introduction

The development of smart medicines has arisen for many reasons, including the challenge associated with using compounds that exhibit poor intrinsic solubility, resistance due chronic use and improving the side effect profile(s) through targeted delivery [1]. Many of these shortcomings can be overcome using nanotechnology, which is defined as the engineering and manufacture of materials at an atomic or molecular scale, resulting in the production of nanoparticles [2] that are broadly defined as materials with dimensions <1000 nm [3,4,5].

**Figure 1:**
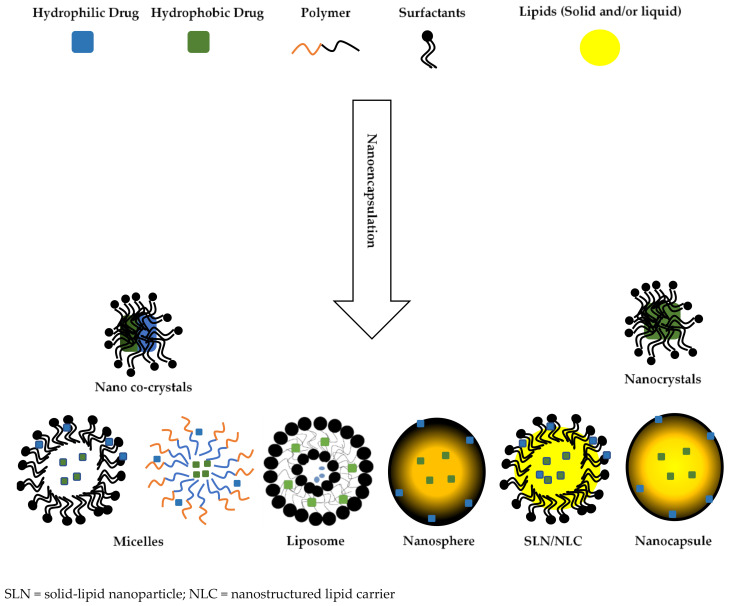
Schematic representation of the different nanoparticles and biomaterials used.

Nanocrystals and micelles are examples of smart nano-drug delivery approaches used for the enhancement of solubility of many active pharmaceutical ingredients (APIs) [6,7,8]. More specifically, nanocrystals of itraconazole [9,10], clarithromycin [11,12,13], luliconazole [14] and micelles of curcumin [15], gliclazide [16] and glibenclamide [17] have been manufactured to improve the intrinsic solubility of these compounds. Liposomes [18,19], nanocrystals [20,21,22], micelles [23,24,25], nanospheres and nanocapsules [26,27,28], solid lipid nanoparticles (SLNs) and nano-lipid carriers (NLCs) [29,30,31] have all been used in attempts to improve the side effect profile of APIs by targeting drug delivery and enhanced stability by shielding APIs from the harsh, gastrointestinal tract (GIT) environment. Nanoencapsulation can be defined as the inclusion of bioactive or the entrapment of natural compounds in carriers that are of nanoscale dimension [32]. The manufacture of the nanoparticles involves the use of biomaterials such as surfactants and/or polymers that form the encapsulation envelope, and in some cases, nanoparticle formation requires the inclusion of surface charge inducers and/or target ligands to achieve the a priori defined, target critical quality attributes (CQAs). Surface charge inducers can increase stability [33], offer cell targeting [34] and muco-adhesion characteristics [35] of carrier technologies. Biomaterials can be any material, natural or man-made, that comprises the whole or part of a living structure or biomedical device which is used to perform, augment and/or replaces a natural functioning element [36]. Characteristic types of nanoparticles and the respective biomaterials used to form the carriers is depicted schematically in Figure 1. 

The selection of an approach for nanoencapsulation requires the consideration of many factors, including the need for targeted delivery, the duration of action required, and in some instances, the potential toxicity profile. When considering nanoencapsulation, further decisions with respect to selecting the appropriate and relevant biomaterials that facilitate the production of nanomaterials with targeted CQAs, including, but not limited to, the particle size (PS), polydispersity index (PDI), Zeta potential (ZP), entrapment efficiency (EE), loading capacity (LC) and in vitro dissolution profile are required. However, in many instances meeting the target CQAs may not directly translate into the production of a clinically relevant dosage form, as the final product may not be biocompatible and consequently not meet the pre-defined quality target product profile (QTPP) for a specific clinical application. Biocompatibility, in its broadest sense, is defined as the interaction of a (bio)material with an appropriate host and the subsequent assurance of that response relevant to the specific application is achieved [37]. More recent definitions aim to describe the biological mechanism of response in more detail [38]. Biocompatibility is evaluated in vitro and in vivo using cell cultures to determine the cytotoxicity of the nanomaterials in vitro, and the administration of the nanomaterial to live animals, usually mice, to evaluate potential carcinogenesis, genotoxicity, immunogenicity and thrombogenic responses in vivo [39]. The complexity of responses observed in a host is the consequence of a series of temporal and spatial conditions involving interdependent mechanisms of biomaterial–tissue interactions that control the ultimate performance of a biomaterial in the biological environment [39]. 

The degree to which a nanomaterial is considered biocompatible is influenced by the host, the properties of the biomaterials used to synthesize the nanomaterial, in addition to the site and duration of exposure of the host to the nanocarrier [40]. The CQAs of the nanomaterials such as the PS [41], ZP [34,42], surface area, concentration and the dose of the payload [43] may also play a significant role with respect to the biocompatibility of the nanomaterials following administration. 

Here, we provide a critical review of the biomaterials used for nanoencapsulation activities and evaluate their putative role with respect to the biocompatibility of the final product, and the overall contribution to the QTPP of the product in order to inform improvement and enhance the application of nanotechnology in modern drug delivery systems. 

## 2. Nanomaterial–Host Interactions 

Following the characterisation of the physicochemical properties of nanomaterials, in vitro testing using cell culture models to evaluate biological effects are required, prior to a full investigation of the in vivo application of the dosage form. Currently used in vitro toxicity tests are not ideal, as there is a lack of phenotypic detail, physiological function or partial elucidation of complex communication processes between cells in addition to poor integration into a tiered approach. Prior to discussing specific toxicity tests, it is prudent to summarize the range of in vitro and in vivo approaches routinely used to evaluate the performance of nanomaterials.

### 2.1. In Vitro Approaches

The initial step towards understanding how a material may interact with a host in vivo requires application of cell-culture testing. When compared to animal studies, cell culture approaches are ethically sound, easier to control, more reproducible and are not as expensive [44].

In vitro cytotoxicity studies of nanoparticles using different cell lines, and incubation times with the aid of a colorimetric or other analytical approach, are necessary to establish the preliminary safety profiles of materials and products [44,45] and such studies have been used to demonstrate the relative safety of nanoparticles, albeit in vitro. While different approaches have been used to determine the degree to of biocompatibility of nanoparticles, it is generally agreed that in vitro testing be categorized as either two- or three-dimensional cell culture models.

#### 2.1.1. Two-Dimensional (2D) Monolayer Cell Culture

Two-dimensional (2D) cell cultures are essentially monolayer cell cultures grown on a plate or the surfaces of a flask that has been treated using physical methods to ensure adherence or makes use of adhesive biological materials to facilitate cell attachment in a specific plane. The cells are bathed with a culture medium, supplemented with nutrients and are maintained at 37 °C in a humidified environment, which provides uniform exposure to oxygen and carbon dioxide, as the minimum requirements for ensuring and maintaining cell viability. The convenience of the monolayer cell culture model is beneficial for the rapid determination of cellular uptake and the intracellular trafficking behaviour of nanomaterials, the bioactivity of APIs delivered using the nanomaterials and the toxicity of the vehicles used. Such studies are usually undertaken with multiple, well established cell lines such as an epidermal cell line A431 [46] or an immortalized human keratinocyte cell line HaCaT [47]. 

##### Cellular Uptake

Confocal microscopy and flow cytometry are widely used approaches for the study of the cellular uptake of nanocarriers which requires the nanocarrier to be labelled with a fluorescent marker and is prepared by physical entrapment or the covalent conjugation of the marker with the carrier. Lipophilic dyes, if used, may leach from nanocarriers on contact with amphiphilic or lipophilic materials, which may confound the assessment of the success of using such drug delivery vehicles [48]. If the intention is to track the passage of a vehicle, it is desirable to label the material through covalent conjugation with a dye and confirm the stability of the conjugation in solutions prepared to be of a similar nature to that of physiological fluids. Ideally, the APIs and vehicle should be labelled separately, to ensure the accurate characterisation of the impact of the vehicle on the delivery process. The potential of 10 nm silica with no surface modification and 30 nm polystyrene nanoparticles with carboxyl surface modification as theragnostic agents for the treatment of ovarian cancer has been reported [41]. In another study, based on the encapsulation of 4-methylumbelliferyl phosphate (MU-P), a profluorophore of 4-methylumbelliferone (MU), liposome tracing is reported [49]. MU-P is rapidly dephosphorylated by endogenous phosphatases in vivo to form MU, following leakage from liposomes. The change in fluorescence spectra, when MU-P is converted to MU, allows for quantification of the entrapped (MU-P) and that which has been released (MU) from the liposome, by sensitive high-performance liquid chromatography analysis and/or fluorescence spectroscopy [49].

Confocal microscopy permits the identification of the location of nanoparticles within cells, however, the quantitative analysis of nanocarrier uptake requires the use of flow cytometry where cells in suspension are passed through a point of detection and are individually examined with respect to their optical or fluorescent properties with the aid of a laser light source [50]. Quantitative information is acquired based on the number of fluorescent cells or the average intensity of fluorescence of the cell population in order to determine the fraction of cells destroyed by therapeutic moieties or the amount of APIs that has been internalised by cells.

The mechanism by which nanocarriers enter cells is as important as the quantity that is internalized, as subsequent intra-cellular events are impacted by the uptake pathway, which is affected by the physicochemical properties of the nanomaterial that are known to enter cells via different pathways [51,52,53,54]. Particles of sizes ranging between a fraction of a µm to ∼10 μm are subject to phagocytosis [51,55], whereas smaller nanocarriers may be transported by micro-pinocytosis [51,55,56], clathrin- and/or caveolae-mediated endocytosis [51,53,57,58,59]. Cells are able to internalise nanoparticles up to approximately 300 nm in diameter by macro-pinocytosis where the cell membrane protrudes and fuses with another part of the membrane to produce relatively large vesicles, in which the particles are enclosed [60]. Clathrin-mediated endocytosis is a consequence of the polymerisation of clathrin proteins located in the cell membrane which then form vesicles of approximately 100 nm in diameter around particles, and after which they are transported as early endosomes [51]. Some nanoparticles may be taken up via the caveolar route [58], where cell membranes coated with caveolin, cholesterol and lipids form flask-shaped invaginations or caveolae to engulf the particles [51]. In particular, clathrin- or caveolae-mediated endocytosis requires specific ligands for cellular receptors [56] such as folic acid [61], transferrin [62] or albumin [63] which facilitate the endo- or transcytosis of these molecules. For this reason, nanoparticles incorporating such ligands have been explored as a means of achieving the cell-specific targeted delivery of therapeutic agents.

##### Intracellular Trafficking

Following internalization, the intracellular fate of nanocarriers will have an impact on therapeutic outcomes, particularly when the drug target is localized in an organelle and/or is unstable in a specific intracellular environment, such as an acidic pH, or is unaffected by the presence of enzymes such as lysozyme, in late end lysosomes. To monitor the intracellular trafficking of nanocarriers, markers for intracellular organelles are co-localised and monitored over a period of time or alternatively, organelles are located using fluorescence via labelled antibodies following the fixation and permeabilization of cells [64,65].

##### Bioactivity

When developing a nanocarrier technology for drug delivery, it is of utmost importance that the potency and efficacy of the payload remain unchanged and/or the specificity of the payload is enhanced due to the use of the technologies. Bioluminescence assays are used to quantitate the ATP produced by living cells using luciferase that metabolizes luciferin in an energy-dependent manner as luciferase activity is proportional to the amount of ATP present and is an indication of cell viability [66]. Dye/stain exclusion assays make use of trypan blue, propidium iodide and calcein-AM which are selectively excluded from or trapped in living cells according to the integrity of membranes or esterase activity. Lactate dehydrogenase (LDH) assays reflect the integrity of cell membranes as LDH is a constitutive cytoplasmic enzyme, which is released when cell membranes are compromised and LDH activity is indicative of the proportion of non-viable cells present at any time [67].

#### 2.1.2. Three-Dimensional (3D) Cell Culture

The use of 2D cell cultures in vitro results in target cells being directly and uniformly exposed to nanocarriers for the specified exposure time without a limit with respect to the maximum concentration in the range to be studied. However, this approach is unlikely to be an accurate reflection of the likely events in vivo when dealing with 3-dimensional (3D) masses, such as solid tumours, where multiple barriers must be overcome prior to the nanocarrier accessing the target cells [68]. Biological barriers include the vasculature and the mononuclear phagocyte system (MPS), cellular barriers such as the cell membrane and specific conditions of the tumour microenvironment such as blood flow and pH [69]. Furthermore, due to unnatural geometric and mechanical properties, 2D-cultured cells have limited potential to represent the phenotypic and genetic function of living tissues, which can and does affect the cellular responses to chemical stimuli [70,71,72,73]. The fact that efficacy in vivo is often vastly different to the screening results generated using 2D cell culture systems is, in part, related to the somewhat artificial nature of 2D cell culture models [74]. Consequently, efforts to develop 3D cell culture models, which better mimic the cell–cell and cell–extracellular matrix (ECM) interactions usually observed in living organisms, have been made in order to ensure a relevant system for the evaluation of drug delivery systems in the nanometre dimension range are available. 

Commonly used 3D models include encapsulated cells in scaffolds, multicellular spheroids [75], combinations of spheroids and scaffolds [76], multilayer cell models [74], excised tissues or tissue components [77] and microfluidic-based devices using a polydimethylsiloxane template [78,79]. Multicellular spheroids are the scaffold-free self-assembled spherical aggregates of cells displaying an intermediate complexity between 2D in vitro cell cultures and in vivo solid tumours, with which they share important similarities [80].

Different models developed for applications, dependent on the route of administration, site of action and targeted toxicity profile are available and include validated topical 3D models such as the EpiDerm™ skin model [81], EPISKIN™ [82], 3D liver cell co-culture containing hepatocytes, hepatic stellate cells, endothelial cells and Kupffer cells [83] and the quadruple culture containing epithelial and endothelial cells, macrophages and mast cells [84]. Other commercially available 3D models include EpiAveolar™, EpiCorneal™, EpiAirway™, EpiIntestinal™ and MelanoDerm™ [85].

### 2.2. Ex Vivo Systems and Evaluation

An alternative promising approach that provides a better in vivo type environment makes use of precision-cut slices of tissue (PCS) that are considered an ex vivo model for the organ of study whilst maintaining the original architecture, i.e., they contain all the cell types of a tissue in a natural conformation. Ex vivo tissue culture models are located between in vitro and in vivo experimental activities [86], in which whole tissue slices such as organotypic slice cultures are used [87]. Ex vivo models based on tissue slices retain the original cytoarchitecture with many intercellular connections and activities intact. Consequently, metabolic processes are more closely representative of the in vivo scenario and may produce results that are most likely to mimic the behaviour in actual use in vivo. Ex vivo studies may be performed using blood samples and/or the cultivation of lymphocytes and have also been applied to the testing of corrosive ocular materials and bovine corneas harvested from slaughtered animals [88]. 

The advantage of using tissue slices from different species is that inter-species comparison is easily achieved using the tissues sourced from biopsies and progress in this area of culture use has been made in the field of pulmonary research [89]. Although most studies have focused on pharma-toxicology [90,91], data proving PCS-based approaches are highly relevant for risk assessment in terms of inflammation, organ injury and the sensitization of nanomaterials and xenobiotics, in general, have been published [92,93,94]. 

The use of co-culture systems including different cell types is possible, further resembling in vivo testing and can be constructed to resemble natural barriers to investigate the mechanism(s) of nanomaterial uptake.

### 2.3. In Vivo Systems and Evaluation 

The demonstration of the proof of concept performance of nanocarriers in vitro is followed by safety and therapeutic efficacy evaluation in animal models. Data generated using animal studies are pivotal in decision making, particularly when establishing whether to progress the technology in question to large-scale multi-centre clinical trials. An animal model in which the pathophysiology of human disease is reflected is necessary and invaluable for the successful translation and prediction of subsequent therapeutic outcomes in humans [95,96]. 

The Organization for Economic Cooperation and Development (OECD) guidelines recommend oral toxicity, eye irritation, corrosion and dermal toxicity testing in addition to the assessment of the LD_50_ to establish the in vivo toxicity of nanomaterials [97].

Colloidal nanomaterials were administered at a maximum oral dose of 5000 mg/kg body weight (LD_50_) to assess and evaluate acute toxicity, after which the animals are monitored at 3 and 24 h for symptoms of toxicity whilst recording the number of surviving animals. The animals are then checked every 24 h over 14 days for symptoms such as oedema, erythema, ulcers, bloody scabs, discoloration and scars in addition to monitoring weight loss, water and food consumption, behaviour. Skin biopsies are performed following 1, 7, and 10 days exposure for histopathological evaluation whilst blood is tested for biochemical changes including but not limited to triglyceride, cholesterol, glucose, glutamic oxaloacetic transaminase (GOT) and glutamic pyruvic transaminase (GPT) levels and other haematological pathology. At the end of the 14 day test period the animals are sacrificed and their skin and liver collected for routine histopathological examination [97].

Colloidal nanoparticles were administered in doses of 1.5 and 2.5 ppm to assess acute eye irritation and corrosion [98]. The test procedure used, is outlined in the OECD 405 guidelines. Briefly, 0.1 mL of the colloidal suspension was placed into the conjunctival sac of one eye of the animal and the other eye, used as a control, was treated with 0.1 mL distilled water. At 1, 12, 24, 48, 72 h and then daily for systems of toxicity, post-administration. The reaction of the eye and conjunctiva, cornea and any chemosis were graded using the OECD 405 guideline grading system [98].

When testing acute dermal toxicity, test animals were divided into three groups (n = 3) randomly to include a group administered distilled water, the second 50 ppm and the third 100 ppm of colloidal suspension. All treatment groups received the dose in 300 μL/cm^2^ [99]. Colloidal nanosuspensions were applied to a shaved skin and the treatment area then covered with a dressing held in place using non-irritating tape for 24 h after which the dressing was removed, and the treatment area gently washed with isotonic saline. The surviving animals were observed every 24 h over a 14-day test period for oedema, erythema, ulcers, bloody scabs, discoloration, scars, weight loss, water and food consumption. Skin biopsies were performed and subjected to histopathological evaluation on days 1, 3, and 7 following exposure and all animals were sacrificed at the end of the 14^th^ -day observation period and the dermatological samples harvested for routine histopathological examination [99].

The acute toxicity of colloidal silver nanoparticles was investigated following the oral administration of a maximum dose of 5 mg/kg and the 14-day monitoring period revealed no mortality or signs of toxicity. All haematological, biochemical and histopathological analyses revealed no difference in any groups examined. The administration of silver nanoparticles at a dose of 5 ppm resulted in transient eye irritation for 24 h and topical application to the skin revealed no macroscopic or microscopic toxicity [100]. 

Gold nanoparticles functionalised with natural compounds extracted from native plants of the Adoxaceae family, specifically the European cranberry bush, *Viburnum opulus* L. and the European black elderberry, *Sambucus nigra* L. exhibited no dermal toxicity [101] as no significant changes in the haematological, biochemical, and histopathological results were observed following the analysis.

It is crucial to understand and appreciate the biodistribution of nanomaterials and these can be monitored following the conjugation of the test material with a fluorescent label or organic dye that can be tracked in blood, tissue and different organs at predefined times following initial exposure. However, there are limitations with respect to the reliability of using this approach, due to the limit of detection of analytical systems when monitoring dye degradation in extended or long-term studies or due to interference when monitoring markers by metabolism and/or the transformation of the nanomaterials into intermediate compounds [97]. This is a common phenomenon with metallic nanoparticles and it has been demonstrated that metabolism and transformation into different oxidative states results in the production of reactive oxygen species (ROS) and subsequent cell death [97]. 

The analysis of biomarkers as a consequence of inflammation and oxidative stress would enhance the mechanistic understanding of nanomaterial toxicity. A test period of 12 and 24 months is currently recommended in the OECD guidelines for the evaluation of chronic toxicity and the carcinogenic potential of nanomaterials after which the survival rate, clinical toxicity, animal behaviour, tumour incidence and histopathological findings in the major organs such as the liver, spleen, heart, kidneys, brain, ovary, and testes can be assessed [97].

## 3. Biocompatibility and Toxicology Testing in Nanotechnology 

A number of different types of toxicity can be tested using in vitro models, which are preferred to in vivo approaches as in many cases, the results are more reproducible and predictive. Carcinogenicity, immunotoxicity, pro-inflammatory responses, genotoxicity and cytotoxicity are investigated in vitro [102] and the biocompatibility described and correlated in terms of the significance and relevance to in vivo data from published research are reported herein.

### 3.1. Immunotoxicity

A limited array of in vitro immunotoxicity tests are available and conventional immunotoxicity approaches do not always detect the true potential for immunotoxicity. Two important general issues with respect to the use of in vitro immunoassays include in vitro sensitivity to nanomaterial-mediated toxicity, and the need for the selection of an appropriate concentration for the test in order for the results to be truly predictive of in vivo toxicity must be addressed [103]. The human-based skin explant assay is a novel tool for the evaluation of immunotoxicity, including adverse immune reactions to chemicals and small molecule drugs and can be adapted to testing the potential immunotoxicity of nanomaterials and nanomedicines [104,105]. For the purposes of this review, the term immunotoxicity includes haemotoxicity.

One aim of using in vitro testing is the rapid generation of data to evaluate the potential of a formulation to precipitate acute reactions in vivo. With respect to the immunotoxicity of nanomaterials, it is generally accepted that if nanomaterials come into contact with blood, the impact on erythrocytes and the complement system must be established in addition to the identification of potential and severe acute toxicity reactions, such as haemolysis and anaphylaxis irrespective of whether the nanomaterial is a component of a medical device, drug carrier, drug or imaging agent [106,107,108,109,110]. Furthermore, the potential ability of a nanomaterial to elicit a thrombogenic response is particularly important when the precipitation of vascular thrombosis and/or disseminated intravascular coagulation (DIC)-like toxicity is likely [111].

The preclinical screening for immunotoxicity is complex as multiple endpoints including platelet, coagulation factors, leukocyte and endothelial cell responses are involved. The plasma protein binding capacity of nanomaterials is widely accepted as an indicator of the rate at which the material can be cleared from the systemic circulation and of distribution of the mononuclear phagocytic system (MPS) [106,111,112,113,114]. The induction of pro-inflammatory cytokines is considered a surrogate measure of cytokine-associated toxicity, including, but not limited to, DIC, pyrogenic potential and hyper-cytokinaemia for which the common markers of acute nanoparticle toxicity include haemolysis, complement activation, thrombogenicity, phagocytosis, pyrogenicity and cytokine induction. Most toxic responses can be rapidly assessed in vitro prior to undertaking more resource- and time-consuming in vivo studies. While the immune responses are initially investigated, the evaluation of potential immunosuppression is vital and can be evaluated using assays that make use of multiple immunological endpoints, with phagocytosis and leukocyte function being the most widely monitored [103].

In vitro pro-inflammatory responses are frequently assessed using reverse transcription polymerase chain reactions to detect pro-inflammatory gene expression in reporter cell lines, and may be used alone or in parallel for enzyme-linked immunosorbent assays [102,115]. Pro-inflammatory cytokines or protein signals of inflammatory responses include monitoring IL-1b, IL-6, TNF-α and chemokine IL-8 activity [116]. Detection is achieved using an enzyme-linked immunosorbent assay (ELISA), which involves quantitation by measuring absorbance due to alkaline phosphatase or streptavidin–horseradish peroxidase-labelled antibodies at 405 or 620 nm, respectively [117].

Complement activation leads to the release of a number of split complement products, some of which are highly reactive and promote an inflammatory response due to C3a, C4a and C5a which are cytokine-like molecules or anaphylatoxins that precipitate anaphylaxis when activated. Complement activation-related pseudo-allergy (CARPA) syndrome is a common dose-limiting toxicity produced when PEGylated-liposomes, other lipid- and polymeric-based nanocarriers are administered [118,119]. Different species of animal exhibit variable sensitivity to complement-activating substances. For example, the total dose of phospholipid sufficient to trigger complement activation related to hypersensitivity reactions is 0.01–0.2 mg/kg in humans, 0.01–0.3 mg/kg in pigs, 0.05–0.1 mg/kg in dogs and 5–25 mg/kg in rats [118] suggesting that pig and dog models may be better in vivo predictors of complement-mediated hypersensitivity reactions in humans than in rat models. Human and non- human primate matrices were found to be sensitive to complement activation when tested against engineered nanomaterials using in vitro complement activation assays, whereas matrices from rat, mouse, mini-pig and guinea pig models were not [120].

Many methods exist for the in vitro determination of complement activation analysis including haemolytic, ELISA-kits, enzyme immuno- and 2D immuno-electrophoretic assays [121] for which each approach exhibits pros and cons and the selection of a particular test method is, in part, dependent on the nanomaterial under investigation as some materials can interfere with certain types of assay or may be used due to the availability of reagents and instrumentation in specific laboratories.

Cytokines are biomarkers of acute inflammation [122,123,124] and IL-1β and IL-6 are indicative of a pyrogenic response. Notwithstanding the untoward responses of nanomaterials, they may be intentionally engineered to elicit or promote immune response by the activation of cytokine expression which makes this property potentially useful for vaccine development activities [125]. In contrast, the undesirable induction of cytokine responses may result in the overstimulation of the immune response resulting in life-threatening conditions such as DIC and cytokine storm. In some instances, the in vitro screening results of nanoparticle-mediated cytokine response have been correlated with in vivo cytokine induction [126]. Two metal oxide nanoparticle formulations (NP1 and NP2) manufactured with identical cores and different surface modifications were tested in a rat and rabbit model, and both nano-formulations produced undetectable endotoxin levels when assessed using a gel-clot limulus amoebocyte lysate (LAL) assay. Formulation NP1 was considered non-toxic, whereas formulation NP2 resulted in animal death. Following histopathologic and necropsy examination, congestion in the spleen and other organs, similar to that observed in septic shock, was evident. The analysis of plasma samples from the affected animals revealed the presence of high levels of the inflammatory cytokines IL-1, TNF-α and IL-8. The administration of formulation NP2 precipitated a cytokine storm in vivo and activated cytokine activity in a normal human peripheral blood mononuclear test model (PBMC) in vitro. These results emphasize the importance of in vitro cytokine testing, prior to using the nanomaterials in vivo.

The opsonization of plasma proteins to nanomaterial surfaces results in nanomaterials being “visible” to phagocytes, thereby facilitating removal from the systemic circulation. Phagocytes use multiple routes for the removal of nanomaterials from systemic circulation including complement receptor-, FcγR- and mannose receptor-mediated phagocytosis and macro-pinocytosis, clathrin-, caveolin-dependent and independent pinocytosis [127,128,129,130]. Proteins that facilitate uptake are termed opsonins and include complement proteins and immunoglobulins. Nanomaterials with unprotected surface attributes due to the presence of hydrophilic polymers are able to bind extensively to proteins [111], however, experimental evidence in support of the link between specific proteins, or protein profiles, in the corona and nanomaterial toxicity has yet to be generated. The most commonly reported protein–nanomaterial interactions occur between Pluronic^®^ or Tween^®^ coated 80 SLN [131], liposomes [132], poly(lactic acid) nanoparticles (NP) coated with PEG [133] and proteins albumin, fibrinogen, apolipoproteins, immunoglobulin G (IgG), α1-antitrypsin, α2-macroglobulin and immunoglobulin M (IgM) [131,132,133]. The experimental significance of the total extent of nanoparticle binding to proteins and the biodistribution to the organs of the MPS, circulation time and inflammation at the site of nanoparticle retention have been elucidated [114]. Irrespectively of the in vitro model used, and whether it included primary cell or cell lines and the type of cells used viz., macrophages, monocytes, or monocyte–macrophages, the evaluation of nanomaterial uptake in vitro is a useful and appropriate surrogate for predicting the likelihood of MPS capture, in vivo. Consequently, in vitro assays can be used for preclinical characterisation activities when screening multiple formulations simultaneously, for the identification and selection of lead candidate(s) with low or no MPS retention potential. Conversely, this method can be used to identify particles that exhibit a high retention behaviour for application when capture by cells of the immune systems would be a useful therapeutic strategy [103].

The incompatibility of nanomedicines with the components of the systemic circulation can be monitored using assays with blood and reactions may include thrombosis, coagulation, platelet activation, changes to blood cells and complement activation. The presence of C3a, C5a, Bb, iC3b, C3 and C5 convertase in addition to other complement components can be monitored [134]. Haemolysis or damage to red blood cells may lead to anaemia or other life-threatening conditions, and an early understanding of the haemolytic potential of nanomaterials is essential when assessing biocompatibility. Correlation between the results of in vitro haemolysis assays and in vivo toxicity studies suggest haemolysis as an undesirable and toxic effect that should be avoided [103]. 

The in vitro haemolysis assay involves the use of human [135] or animal blood [136] with the addition of anticoagulants such as potassium oxalate and the subsequent exposure to the nanomaterials under investigation. The selection of anticoagulant, animal species from which the blood is harvested, the protocol used and the extent of haemolysis in vitro are an important consideration, however, the anticoagulant and the origin species of blood are not that critical, as it has yet to be demonstrated that significant differences in assay test results arise when these parameters differ. The American Society for Testing and Materials (ASTM) International protocol E2524-08 [137] is commonly used when investigating the haemolytic properties of nanoparticles as it sets a 2% threshold for in vitro haemolysis [137], indicating if the assay result for a test material is <2%, it is considered non-haemolytic, whereas between 2 and 5% are moderately haemolytic and that >5% reflects that the test nanomaterial is haemolytic. In some studies [112,113,114,115,116,117], nanoparticles not considered haemolytic in vivo exhibited <2% haemolysis in vitro, and materials which caused haemolysis in vivo, exhibited >5% haemolysis when tested in vitro. 

In vitro haemolysis experiments, in general, are reflective of in vivo performance and reports suggest that cationic dendrimer use resulted in 14–86% haemolysis, in vitro, when nanomaterials were tested using whole blood from human donors, different animal species and following the administration to rodents, decreases in the erythrocyte count, haemoglobin and haematocrit levels were observed [138,139,140,141,142,143]. In vivo toxicity studies of nanoparticles that exhibited in vitro haemolysis between 4 and 5% resulted in a decrease in the erythrocyte and haemoglobin counts, in addition to lowering the haematocrit values in all animals tested [144] and the nanomaterials that exhibited in vitro haemolysis >50% resulted in the immediate death of animals when administered intravenously [144].

### 3.2. Genotoxicity and Carcinogenicity

Genotoxicity is an important evaluation to be conducted when testing nanomaterials and assays may have different endpoints, including single- and double-strand breaks, mutation, deletion, chromosome aberration, micronucleus formation, DNA repair and/or cell-cycle interactions [145].

It is important to include the genotoxicity test when assessing biocompatibility, and the most commonly applied approaches to detect the genotoxicity of bulk chemicals include the bacterial Ames test, DNA strand break measurements in cells including the comet assay, alkaline unwinding and hydroxyapatite chromatography, in addition to alkaline elution. Cytogenetic assays such as micronucleus (MN) and chromosomal aberration testing, in addition to in situ fluorescence hybridization and chromosome painting, have also been applied to the evaluation of genotoxicity [127]. 

The mammalian cell gene mutation assay, or the adaptation of the mammalian cell micro-nucleus assay is used to detect chromosome breakage that leads to the formation of an additional nucleus or micro-nucleus during cell division [115,146].

More extensive cytotoxicity studies have been used to establish the genotoxic potential of nanomaterials by examining the extent of DNA damage and one extensively used approach evaluated the effect of exposure to carbon nanoparticles by flow cytometry [147,148,149,150] in which a laser beam that differentiates cells based on size and density is applied. With the aid of DNA intercalating dyes, cellular DNA can also be used to determine the apoptosis of cells which occurs as the dye binds to nucleic acid, the extent of which increases, as membrane permeability increases [151]. The comet assay has been used to detect DNA damage in individual cells using gel electrophoresis, as damaged DNA appears as “comets” with intact DNA residing in the head portion and broken DNA pieces migrating away from that head, forming a tail. A DNA-specific dye, propidium iodide is used to read the gel and the amount of DNA found in the tail is generally proportional to the extent of DNA damage that occurs [152].

The H2AX assay is used to quantitate breaks in double-strand and is based on the fact that a cellular response to these breaks includes the phosphorylation of one of the nucleosomal histones, H2AX, in the core. The concentration of the phosphorylated form of the peptone, γ-H2AX, in proximity to a double-strand break is sufficient to form a focus which is visible using immune-histochemistry and a fluorescence-tagged antibody that binds to the phosphorylated compound, Alternatively, γ-H2AX can be quantitated using flow cytometry. 

Mammalian gene mutation assays are also suitable for establishing the toxicity of nanomaterials as they detect a range of mutations from point gene mutations to small and large deletions of genetic material. The most commonly used tests make use of *HPRT* or the *TK+/*− locus which, in the mouse lymphoma assay, detects deletions with higher efficiency when compared to the *HPRT* gene mutation test. HPRT is a purine salvage enzyme, which phosphorylates “waste” purines and then adds them to the cellular DNA precursor nucleotide pool. The *HPRT* gene is X-linked, with only one active copy per cell, indicating that a mutation in only one allele is required to ensure phenotypic expression [145]. Recently, the in vitro phosphatidylinositol glycan anchor biosynthesis, class A (Pig-a) gene mutation assay has been added as an in vitro mammalian mutagenicity test tool [153]. 

The MN assay can be applied to in vitro and in vivo testing and has been recommended for assessing the mutagenic and clastogenic effects of pollutants [154] as it can easily be used to evaluate different target cells and tissues [154,155] and the assessment of the nano-toxic potential of compounds [145,156]. For micro-nuclei formed from the residual fragments of whole chromosomes following nuclear division, during anaphase and following telophase, the fragments may not be included in the nuclei of daughter cells but rather form single micro-nucleus or multiple micro-nuclei in the cytoplasm of the cells [154]. The MN test is used to detect the clastogenic and aneugenic effects [154] whilst detecting the genotoxicity of a wide range of compounds, including nano-materials. The formation of nucleo-plasmic bridges and binucleated cells results in the complementary detection of chromosome rearrangement. The sensitivity of the assay can be increased by the addition of cytochalasin-B, a cytokinesis-blocking agent that inhibits cell division or the use of differential staining methods such as fluorescent acridine orange which is now a routine measurement approach.

The genetic instability of gross chromosomal abnormalities or comparatively minute aberrations such as single base-pair substitutions are associated with carcinogenesis and have been detected in all malignancies [157]. It is widely accepted that the cancer phenotype resulting from the accumulation of multiple genome-wide mutations drives tumorigenesis, in a multistep fashion which progressively transforms normal healthy cells into premalignant foci, which subsequently become localized tumours that develop further into invasive and metastatic lesions [158]. There is a general paucity of in vitro tests for the assessment of the carcinogenicity of malignancy-forming compounds. 

The most in vitro test for carcinogenicity makes use of the cell transformation assay (CTA) which evaluates the potential cell transformation and has an ability to detect both genotoxic and non-genotoxic carcinogens. CTA is a relatively new approach used to evaluate carcinogenicity [159] and The European Union Reference Laboratory proposed, in 2015, in the guidance document titled “In vitro Syrian hamster embryo (SHE) cell transformation assay”, that this approach be used to evaluate the carcinogenic potential of substances [160,161]. The availability of the CTA, in which primary cell cultures such as the Syrian hamster embryo cell and/or BALB/c 3T3 cell or genetically modified Bhas 42 cell lines, are incubated with test compounds after which the treated cells are analysed to determine expression of the traits associated with tumour development in vivo and have subsequently become the cornerstone of carcinogenicity testing [159].

### 3.3. Cytotoxicity

Cytotoxicity, the cornerstone of in vitro toxicity testing, is an assessment of cell viability and is a necessary test for all nanomedicines and component biomaterials [115]. Cell lines of different origin, such as intestinal or pulmonary cells, for example, can be used to predict acute toxicity by the estimation of the lethal concentration to 50% of the exposed cells (LC_50_ values) using 2D and 3D systems that target the specific endpoints of testing [127].

Cytotoxicity testing assesses cell damage due to intracellular changes, changes in cell proliferation, cell metabolism, membrane integrity, cell morphology, necrosis and apoptosis. It is important that an appropriate cell line be selected for use whilst balancing the intended application of the drug delivery technologies under evaluation and the characteristics of the test used to evaluate cytotoxicity [115]. 

Cell viability can also be assessed using compromised cell membrane integrity assays which evaluate the leakage of cellular content. Most cytotoxicity tests are colorimetric assays that make use of the optical activity of organic dyes [162] including neutral red uptake (NRU), trypan blue (TB) and lactate dehydrogenase (LDH; cytosolic enzyme) release assays [163]. Neutral red dye is weakly cationic, readily diffuses across membranes and accumulates in cellular lysosomes. Increased cell permeability and lysosome fragility are associated with the end stages of apoptosis and the level of dye in the cells is an indicator of cell viability. The NRU assay, is more sensitive than other cytotoxicity tests and dye uptake is easily quantitated using a plate reader and as internalized nanomaterials can be sequestered in lysosomes, an increase in lysosomal activity does not always reflect a reduction in cell viability. It is important to note that different endpoints are used to reflect distinct metabolic functions or a lack thereof, and assays differ significantly in terms of sensitivity [127]. The trypan blue exclusion assay is suitable for identifying damaged cells only, as the dye is only taken up by damaged cells, and since the assay detects cell membrane damage, it is not necessarily a reflection of cell death [127]. The LDH assay measures cellular enzyme activity as damaged cells release LDH and is also a measure of cell membrane damage and not necessarily total cell death [127]. The results are affected by the number of cells used in the test and any inhibition of proliferation that occurs on exposure and reflects a decrease in cell concentration must account for this inhibition to avoid an overestimation of cytotoxicity. 

The outermost surface of healthy cells is made up of asymmetrically distributed lipids on the inner and outer leaflet of the plasma membranes. Phosphatidylserine (PS), a main lipidic component of the plasma membrane, is normally restricted to the inner leaflet of the plasma membrane and is, therefore, only exposed to the cytoplasm of the cell. However, during apoptosis, lipid asymmetry is lost, and PS is exposed on the outer leaflet of the plasma membrane, allowing Annexin-V, a 36-kDa calcium-binding protein, to bind to PS. Consequently, fluorescent-labelled Annexin-V is used to detect the PS located on the outside of apoptotic cells. In addition, Annexin-V stains necrotic cells since the cells have ruptured membranes that permit Annexin-V to access the components of the entire plasma membrane. In order to distinguish apoptotic and necrotic cells, co-staining with propidium iodide (PI) is included as it can enter necrotic cells but is unable to access apoptotic cells [164]. 

Tetrazolium salts can be used to evaluate mitochondrial activity, which is a useful measure of cell metabolism since mitochondrial dehydrogenase enzymes cleave the tetrazolium ring, as these enzymes are only functional in inactive mitochondria. Cell morphology can be monitored using phase-contrast microscopy to visualize morphological alterations in living cell units [165].

Traditional cytotoxicity assays lack specificity when applied to nanomaterial testing and recent studies suggest that standard cell-based assays designed for testing chemicals may not produce reliable data since nanomaterials can and do interfere with the components of assays that require optical read-out systems [162,166,167], which is a consequence of the large surface area and high surface energy of nanomaterials that may adsorb assay reagents. Therefore cytotoxicity data should be verified using at least two or more independent test systems [167] to eliminate misleading results and the incorrect interpretation thereof. 

Since nanomaterials can interfere with in vitro test systems and may produce artefacts independent of the potential toxicity of the nanomaterials, there is a need for the development of new in vitro assays using approaches which reduce potential interference. Many in vitro assays make use of dynamic light scattering (DLS) and other light-based detection systems and are therefore not suitable for the analysis of many types of nanomaterials. Digital holographic microscopy (DHM) is a novel non-intensity-based and label-free tool which is used to analyse parameters, such as the refractive index, which can be correlated to protein content, cell thickness and dry mass, in addition to monitoring the cellular dry mass over time [127].

The most reliable assays for the cytotoxicity testing of nanomaterials include the proliferation and clonogenic or colony-forming efficiency (CFE) assay of which the CFE approach has recently been validated for nanomaterial assessment [160]. The CFE assay makes use of single cells seeded at low density, and subsequently counting colonies with a minimum of 50 cells at pre-defined times following the exposure to establish the viability (colony number) and proliferative capacity (colony size) which are then used to establish the LC_50_ [160].

In vivo carcinogenicity testing is based on the daily administration of a test substance in graduated doses to groups of test animals, for the majority of the life span, whilst closely monitoring animals for signs of toxicity and the development of neoplastic lesions.

**Figure 2:**
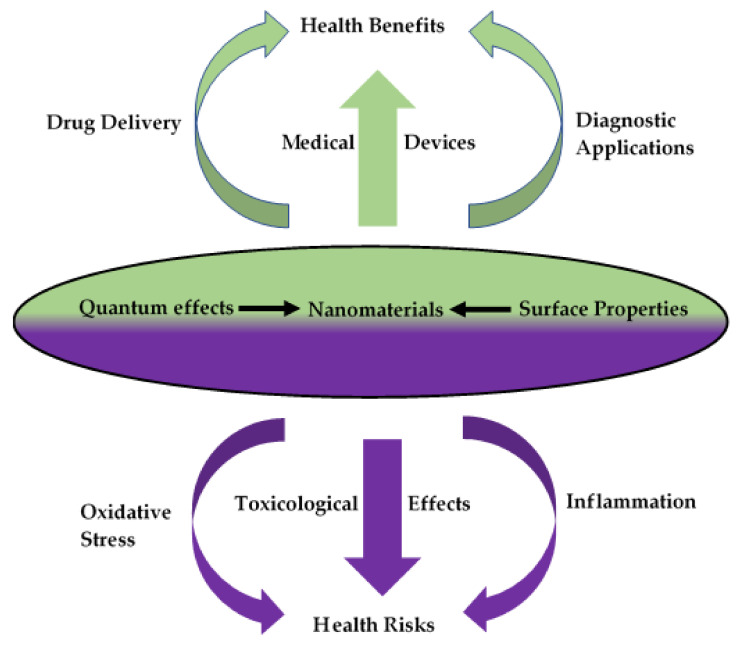
The relationship between nanomaterial properties, applications and potential toxicity (Adapted from [168]).

A schematic diagram showing how surface properties and quantum effects are the properties of nanomaterials responsible for both medical applications and potential toxicological effects is shown in Figure 2 (adapted from [168]).

## 4. Biocompatibility of Biomaterials Used for Nanoencapsulation

### 4.1. Lipids 

Most data generated with respect to the effects of nanomedicines focus primarily on targeted cellular models for which the desired outcome of the development process is the production of a technology that results in a specific therapeutic effect with limited or no side effects. This section explores the biocompatibility of lipids that have been used for nanoencapsulation as it is an important approach for enhancing the bioavailability of encapsulated active substance(s) and provides protection against natural and processing effects, viz., chemical [169], enzymatic and physical instability of nutraceutical [170], pharmaceutical and cosmetic products [169,171]. Furthermore, improved biological and dosing outcomes, due to the controlled delivery of active pharmaceutical ingredients (API), the administration of low doses and the improved shelf-life of encapsulated moieties may possibly reduce the potential emergence of side effects [172]. There are several reports with respect to the biocompatibility of some lipid excipients used for the nanoencapsulation of different molecules, some of which are summarized, *vide infra*. 

The haemocompatibility of nanoparticles is of critical importance when the direct and/or indirect systemic administration of drug delivery systems is to be considered. The haemocompatibility of citrem [173] may be used in combination with soybean phosphatidylcholine (SPC) or Myverol 18e99K, a commercial monoglyceride. to produce a unique range of safe lamellar and non-lamellar liquid crystalline nanoparticles [174]. The authors suggest that citrem eliminates complement activation due to the structural similarity between the terminal citric acid moiety and sialic acid, which results in an inhibitory effect on the activation of the third complement protein (C3) via the interaction of the complement regulatory protein factor, H, with the carboxyl functionality of the citrem molecule [173]. Furthermore, citrem can be accommodated in the internal hydrophobic domain of nanostructures leading in a concentration-dependent manner, to significant structural alteration and phase transition [173,174]. In addition to modulating the internal nanostructure of nanoparticles, thereby providing structural tunability, citrem prevents complement activation which prevents haemolysis [173,175]. The haemocompatibility of poly (ɛ-caprolactone) lipid-core nanocapsules stabilized using polysorbate 80 and lecithin and coated with or used without chitosan exhibited no significant haemolysis or platelet aggregation, suggesting the lipid-core nanocapsules were haemocompatible [176]. Isotonic and sterile lipid-core aqueous suspensions of nanocapsules manufactured using poly (ε-caprolactone), sorbitan monostearate, capric/caprylic triglyceride and glycerol, when incubated with red blood cells with and without glycerol, exhibited no signs of haemolysis at any of the concentrations investigated, suggesting that the aqueous isotonic and sterile lipid nanocapsule dispersions would be suitable for intravenous administration as a nanomedicine [170]. 

Lipid-based nanosystems can be prepared using biocompatible lipids including phospholipids, cholesterol and triglycerides that exhibit advantages such as biocompatibility and biodegradability in addition to lower toxicity in comparison to polymeric nanoparticle drug delivery systems [177]. No evidence of associated toxicity following the administration of biodegradable nanocarrier systems for bioactive compounds exists, however, the health risks usually associated with long-term use requires ongoing investigation [178,179]. The cytotoxicity of albendazole in the free form and encapsulated in SLNs in a U-87 MG human glioblastoma cell line has been reported [180] and the results reveal that the delivery of albendazole using SLNs was cytotoxic to the U-87 MG cells as a consequence of efficient uptake by these cells. In order to conclude that the use of glyceryl trimyristate and polysorbate 80 in the SLNs was a safe control, SLNs were tested without cytotoxic effects indicating that the cytotoxicity of albendazole-loaded SLNs was likely due to the encapsulated albendazole [180]. The development and testing of topotecan-loaded SLNs and NLCs exhibited improved cytotoxicity following the nanoencapsulation of topotecan with control nanoparticles exhibiting little impact on the viability of K-562 cells over the exposure time used [181] and the results further confirm the biocompatibility of stearic and oleic acid. Enhanced cytotoxicity, higher intracellular concentrations and a greater degree of apoptosis was observed when bleomycin NLC particles were tested in a HeLa cell line when compared to bleomycin use alone or control NLC particles using stearic acid that exhibited no effect on cell viability under the conditions investigated [182]. In the validation of in vitro cytotoxicity testing of methotrexate-lipid nanoparticles for the treatment of osteosarcoma, the nanoparticles exhibited different cytotoxicity between the methotrexate used alone and the control particles [183]. All results were statistically significant, however, the encapsulated methotrexate exhibited the greatest efficacy in the osteosarcoma cell line, possibly due to the temporal kinetics involved in the cellular internalization processes of nanoparticles, and not necessarily the lipid used in the formulation, in this case, Precirol^®^ ATO5 [183]. Docosahexaenoic acid-loaded NLCs produced using a blend of Precirol^®^ ATO5, Miglyol^®^ 812 and polysorbate 60 were bactericidal to *Helicobacter pylori* and the NLCs were not cytotoxic to human gastric adenocarcinoma cells at bactericidal concentrations [184]. A cytotoxicity study evaluating the effect of pomegranate extract-loaded SLNs on human breast carcinoma (MCF-7), human prostate carcinoma (PC-3), and human hepatocellular carcinoma (HepG2) cell lines and human normal melanocyte (HFB-4) cells revealed a significant difference in the IC_50_ of pomegranate SLNs and the control SLNs on the MCF-7 cell line [185]. These results were attributed to the effect of stearic acid which has been reported to possess anticancer activity in breast [186] and prostate cancer, specifically the PC-3 cell line [187]. The use of nanotechnology for specific targeting with control nanoparticles resulted in no change in the viability and apoptosis of cells during cytotoxicity testing, suggesting that the lipids used for nanoencapsulation are biocompatible [188,189,190,191,192,193]. SLNs or NLCs are, in general, non-toxic to cell culture lines or in vivo and any toxicity that prevails is dependent on factors such as size, chemical components, capped surfaces or surface charges [194]. It is essential that the safety of these systems be evaluated, on a case-by-case basis, as it is not possible to generalize the activity and behaviour in cells in vitro and/or during in vivo studies. 

Genotoxicity may lead to mutagenic and/or carcinogenic responses in some circumstances [195]. The genotoxicity of lipid nanocapsules undertaken to assess the role of lipid nanocapsule size, composition and surface charge has been reported [192] and the lipid nanocapsules were manufactured using Solutol^®^ HS15 surfactant, Captex^®^ 8000 and Lipoid^®^ S75-3. The genotoxicity evaluated using the comet and micronucleus assay on human lymphocytes, revealed no DNA-damaging lipid nano-capsule concentrations that were not, cytotoxic. Surprisingly, there is a dearth of information and very few studies in which the biocompatibility of lipids has been investigated in terms of genotoxicity, histocompatibility and carcinogenesis therefore, emphasis must be placed on such evaluations in future research activities.

### 4.2. Phospholipids 

Phospholipids are naturally occurring biodegradable excipients that exhibit excellent biocompatibility and are the main components of the cell membranes of eukaryotic organisms which are amphiphilic structures with wetting properties and an ability to self-assemble and emulsify [196]. Phospholipids are used for the formulation of drug delivery systems such as liposomes, emulsions, mixed micelle, phospholipid micelle, drug-phospholipid complexes and phytosomes, amongst others [197,198,199]. 

**Figure 3:**
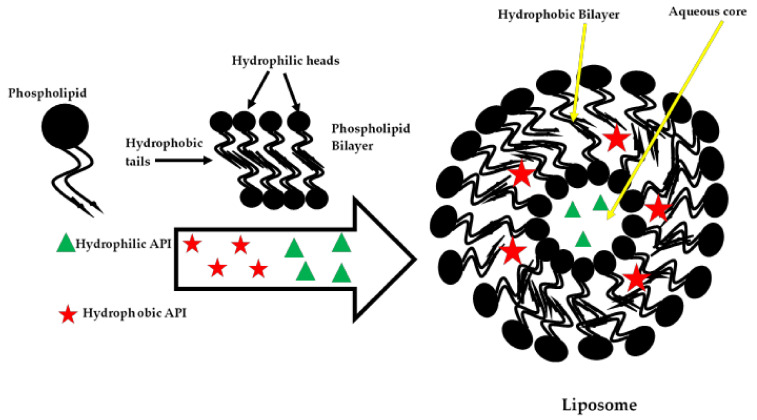
Schematic representation of liposome formation and the encapsulation of active pharmaceutical ingredient (API) molecules.

Self-assembling phospholipid-based nanoparticles (PBNs) of nanometre dimensions, such as liposomes, are popular and have been successfully used in drug delivery research and clinical applications. Liposomes are small spherical-shaped vesicles that range from a few nanometres to several micrometres in size. Liposomes generally consist of one or more phospholipid bilayers that encapsulate an aqueous compartment, as illustrated in Figure 3, making them versatile carriers that can encapsulate materials of different polarity and physicochemical properties [200,201,202]. In the biomedical sphere, liposomes are a most successful delivery system as they have co-loading capabilities and resemble the cell membranes from a structural perspective, are known to be biocompatible, biodegradable, non-toxic and are non-immunogenic carriers that improve drug solubility, offer control over distribution, and the possibility of the targeted delivery of the payload when surface modifications are included [200,203,204,205].

**Figure 4:**
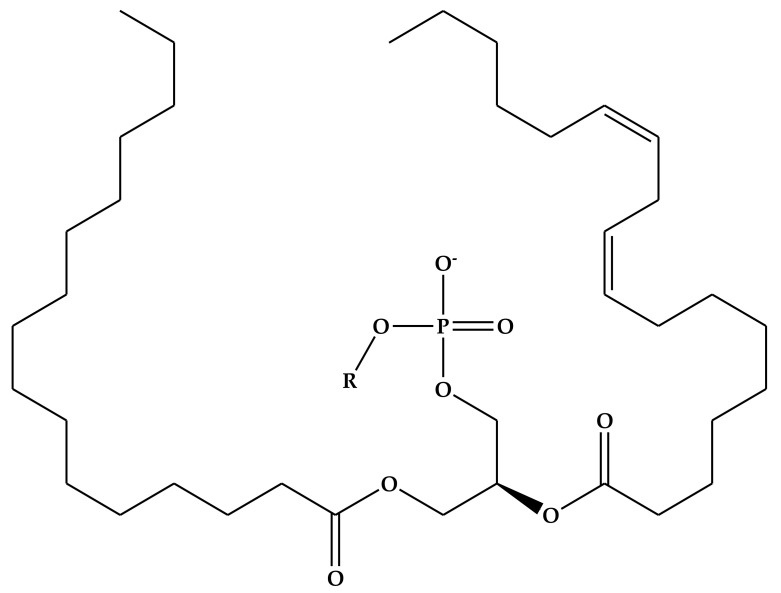
General structure of phospholipids.

The most commonly used natural phospholipids for the preparation of conventional liposomes include phosphatidylcholine (PC), phosphatidylethanolamine (PE), phosphatidylserine (PS), phosphatidylglycerols (PG), phosphatidic acid (PA), phosphatidylinositol (PI), cardiolipin (CL) and sphingomyelins (SM). The basic structure of phospholipids and their charge characteristic depending on substituent structure in a neutral environment are depicted in Figure 4 and Table 1, respectively. 

One challenge of using these materials relates to the purity of natural phospholipids, of vegetable and/or animal origin, which is relatively difficult to control. In addition, natural phospholipids are unstable and form lysophospholipids during emulsification, sterilization and/or storage. For example, lysophospholipids generated from lecithin emulsions have been reported to cause haemolysis following intravenous injection [197,198]. 

Conventional liposomes exhibit some limitations in terms of drug delivery due to systemic clearance by the mononuclear phagocyte system (MPS) [206], which has led to the development of synthetic and semi-synthetic phospholipids and phospholipid complexes for the design of novel PBN that exhibits improved bioavailability and interesting delivery features, such as longer systemic circulation times, stimuli-responsiveness, targeting properties and improved stability [197,207].

Physicochemical characteristics such as surface charge, particle size, lamellarity, physicochemical properties of the payload and the lipid composition of vesicles are known to impact the immunogenic properties of PBN. Cationic liposomes have been identified as being superior when compared to zwitterionic and anionic liposomes, with respect to the stimulation of antigen-presenting dendritic cells (DC) and cationic lipids with ethyl phosphatidylcholine head groups are better stimulants than trimethylammonium-based compounds [208].

Although cationic lipid used in liposomes may promote cellular delivery and induce inflammatory responses, they can also lead to immunotoxicity to some extent [209,210,211]. Use of the LDH assay and differential cell count analysis revealed dose-dependent toxicity and pulmonary inflammation when cationic liposomes were used in a murine model, which suggested that the toxicity observed was related to the charge ratio, since the multivalent cationic liposome LipofectAMINE^®^ had a pronounced effect when compared to the use of a monovalent 1,2-dioleoyl-3-trimethylammonium-propane (DOTAP) cationic liposome [212]. In this case, no toxicity to lung tissue was observed, when neutral liposomes manufactured with palmitoyl-oleoyl phosphatidylcholine (POPC) or negatively charged liposomes made with a mixture of POPC and dioleoyl phosphatidylglycerol (DOPG) were tested [212]. This may be related to the fact that phospholipids, particularly PC and PG derivatives, are primary components of most mammalian lung surfactants and are important for reducing surface tension and maintaining the stability of alveolar structures [213,214]. In a separate study, LipofectAMINE^®^ 2000 liposomes were toxic to mammalian cells whereas CDAN/DOPE liposomes, manufactured with a mixture of cationic cholesterol-based polyamine lipid *N^1^*-cholesteryloxycarbonyl-3,7-diazanonane-1,9-diamine (CDAN) and dioleoyl phosphatidylethanolamine (DOPE), were found to induce low toxicity [215]. The toxicity to normal/healthy cells was explained by the electrostatic interaction between the positively charged liposomes and normal/healthy negatively charged cell membrane surfaces or the generation of reactive oxygen species in cells due to the presence of cationic head functional groups [207].

Neutral lipids can be combined with cationic lipids to exert synergetic effects including the reduction of the in vivo toxicity of cationic liposomes to normal healthy cells in cancer therapy. The use of neutral lipid-based nanoliposomes manufactured with 1,2-dioleoyl-sn-glycero-3- phosphatidylcholine (DOPC) was safe and more effective than when cationic liposomes were used alone for the systemic delivery of siRNA to tumour tissues in a murine model [216]. The DOPE-containing liposomes and cholesterol derivatives have been reported to exhibit low toxicity and high transfection efficiency in human hepatoma cells (Hep G2) [217] that can be explained by the ability of DOPE to increase the hydrophobicity of liposome membranes and the subsequent facilitation of membrane fusion [200,217] that destabilizes plasmalemma or endosomes [217] and the low toxicity may be due to the presence of the cholesterol derivate. The incorporation of cholesteryl arginine ethyl ester (CAE) as a cationic ligand in cationic liposomes containing paclitaxel (PTX)-loaded soy phosphatidylcholine (SPC), revealed slightly improved cytotoxicity in vitro, and endothelial cell migration inhibition when compared to that observed for conventional liposomes made with SPC, cholesterol and PTX. Furthermore, CAE did not exhibit genotoxicity when tested using the COMET assay and both SPC/CAE and SPC/cholesterol-free liposomes had no effect on the migration ability of endothelial cells [218]. 

The size of liposomes is important for vaccine delivery and route of administration. The encapsulation of plasmid DNA (pDNA) into small cationic liposomes manufactured with egg PC, dioleoyl-phosphoethanolamine (DOPE) and DOTAP was suitable for DNA vaccination via subcutaneous injection with the result of robust induction of OVA-specific, functional CD8^+^ T-cells and higher antibody levels, when compared to the use of large hetero-dispersed liposomes and the administration of naked pDNA [219].

The transition temperature (T_c_) of phospholipids or the lamellarity of vesicles that are formed have been reported to affect the immuno-activating potential of liposomes. Liposome-induced proliferative reactions in popliteal lymph nodes (PLNs) has been assessed using a cytometric assay and multilamellar liposomes (MLVs) containing diestearoyl phosphatidylcholine (DSPC) and dipalmitoyl phosphatidylcholine (DPPC), which have a characteristic relatively high transition temperature (Tc), induced an extensive PLN reaction in a dose and time-dependent manner, whereas liposomes manufactured with the lipids of low T_c_ viz., egg phosphatidylcholine, dimyristoyl phosphatidylcholine (eDMPC) did not activate the immune system [220]. Liposomes with identical phospholipid compositions, small uni-lamellar vesicle (SUV) liposomes also exhibited a reduced immune-activating potential when compared to large MLV [220].

The characteristics of phospholipid bilayers may also induce adverse immune reactions by the activation of the complement C system, which is also known as complement (C) activation-related pseudo-allergy (CARPA), in vivo. In vitro and in vivo studies were used to elucidate the contribution to reactogenicity of liposomal delivered drugs, particularly Doxil^®^ and AmBisome^®^ products that include a surface charge and the presence of doxorubicin which is as a shape-modifying drug, resulting from liposome-induced C activation and the associated hypersensitivity reactions. In addition, other parameters, such as choice of PC, length of the carbon chain and the surface density of polymer-phospholipid conjugates exhibit minor or no effects and do not induce CARPA [221]. 

The physicochemical properties of the payload may also have concentration-dependent effects on the biocompatibility of PBN. The haemocompatibility of natural phosphatidylcholine and phosphatidylinositol in liposome formulations containing lipophilic prodrugs viz., methotrexate or melphalan 1,2-dioleoylglyceride esters incorporated into the lipid bilayer of liposomes has been investigated [222]. The melphalan liposomes did not induce undesirable side-effects, whereas the methotrexate liposomes resulted in significant C activation with abnormal coagulation times, in a concentration-dependent manner. Decreased methotrexate loading, from 10 to 2.5% in the liposome, resulted in a lower surface charge density, smaller liposomes and considerably reduced C activation and impact on the coagulation cascade [222].

The main hurdle to nanotechnology, particularly conventional liposome use, is the in vivo recognition and clearance by the MPS of the host [206]. This biological fate suggests these may be suitable delivery systems for the passive targeting of diseases where the target is located inside MPS cells such as leishmaniasis and tuberculosis as any decrease the circulation half-life of nanoparticles may lead to therapeutic failure when the target site of action is external to the MPS [223]. 

The physicochemical properties of phospholipids play an important role in MPS recognition and must be a primary consideration when designing and developing PBN. Liposomes that resemble the exterior layer of erythrocytes, which is PC and SM, are likely to resist clearance by the MPS. Liposomes in which negatively charged phospholipids such as PS, PG and PA are cleared rapidly from the systemic circulation, whereas those containing negatively charged ganglioside and PI generally escape uptake by the MPS. Increasing the rigidity of liposome structures by incorporating phospholipids, such as SM or those with high transition temperatures, such as distearoyl phosphatidylcholine (DSPC), may also reduce uptake by the MPS [197]. 

“Stealth” liposomes are produced by grafting hydrophilic biocompatible polymers such as PEG to phospholipid components and is a strategy used extensively to avoid uptake by the MPS thereby prolonging the circulation time of liposomes and therefore the payload [224]. Liposome-encapsulated haemoglobin formulations manufactured using the sterically stabilizing lipid, polyethylene glycol-distearoylphosphatidylethalonamie (PEG-PE), were less immunotoxic in animals when compared to the use of conventional liposomes, where other lipids, such as PG from eggs, mixtures of distearoyl phosphatidylcholine, cholesterol and dimyristoyl-PG, and PI saturated with egg-PC and cholesterol were used for the encapsulation of haemoglobin [225].

PEGylated liposomes can, however, lose their long-circulating characteristics and may be affected by an accelerated blood clearance (ABC) phenomenon, due to their size and surface charge [226]. The net anionic charge on the phosphate moiety of the phospholipid–mPEG conjugate plays a key role in the activation of classic and alternative pathways of complement and anaphylatoxin production. Anionic PEGylated DPPC vesicles significantly decrease haemolytic activity in serum and increase plasma thromboxane B2 levels in rats in addition to complement activation when removing the negative charge by methylation of the phosphate oxygen of phospholipid–mPEG conjugate [227]. 

Despite the convention that PEG exhibits no or low immunogenicity, it has been reported that anti-PEG IgM is activated following an initial dose of control PEG liposomes in addition to inducing the ABC phenomenon, particularly when administration in the same animal at pre-defined intervals was repeated [226,228]. Nevertheless, a reduction in the particle size and use of PEGylated micelles made with PEG200-PE-PC led to a reduction in anti-PEG-IgM titres, and consequently the administration of a second injection of PEGylated micelles resulted in a different accelerated blood clearance (ABC) phenomenon when compared to the administration of PEGylated liposomes made with PEG_200_–DSPE–cholesterol [229].

The current trend is to explore other polymers for the development of more potent polymer–PBN conjugates in addition to the use of PEG-modified liposomes made with Pluronic^®^, which have exhibited similar “stealth” behaviour as observed with PEGylated liposomes with better treatment outcomes [230,231] and demonstrated biocompatibility in an ex vivo model for haemolysis using human erythrocytes [231]. Recently, the in vitro biocompatibility of liposomes and a novel poly (3-hydroxybutyrate) (PHB) liposome with mammalians cells was investigated and the MTT cell cytotoxicity assay using HEK cell lines revealed a significant decrease in the metabolic activity of the cells in a dose-dependent manner for liposome and PHB-liposome particles. The cytotoxicity test for liposomes made with PC and cholesterol in addition to PHB liposomes was performed using the LDH assay with HEK and HaCaT skin keratinocytes cell lines and the results revealed evidence of low toxicity for both types of particles. The incorporation of the bacterial polymer, PHB, seemed to improve the potential of PHB liposomes, but further studies in which the cytotoxicity and behaviour of liposomes/PHB liposomes in vivo, following the encapsulation of APIs, are necessary [232]. Depending on the surface characteristics, PBN can aggregate or interact with immune cells in vivo and are subsequently cleared from the blood by the MPS or may induce anaphylactic reactions which jeopardize treatment success. The consideration of the biocompatibility of phospholipid nanoparticles or phospholipid complexes is important and must be evaluated to improve the success of PBN therapy.

### 4.3. Polymeric Materials 

Polymeric nanoparticles such as micelles, dendrimers, nanospheres and nanocapsules [233] have found application in scaffold [234,235], controlled/sustained [236] and targeted drug delivery [237]. The most commonly used polymers include naturally occurring compounds such as chitosan [238] and alginates [239] or synthetic polymers such as Pluronic^®^ [240,241,242], poly (ɛ-caprolactone) (PCL) [236,243], poly (DL-lactic acid) (PLA) [244,245], polyethylene glycol (PEG) [133,246,247], Eudragit^®^ [248,249,250,251], poly (vinyl alcohol) (PVA) [252,253], poly lactide co-glycolic acid (PLGA) [254,255] poly (amindoamine) (PAMAM) and poly (propylene imine) (PEI) [256].

Chitosan is a polysaccharide with a chemical structure similar to vegetable fibre cellulose that exhibits antifungal and antimicrobial activity, the inhibition of tumour cell growth and imparts controlled release activity to delivery technologies [257]. Chitosan has been used to sustain the release of APIs [258,259], is a mucoadhesive [260,261,262] and a positive charge inducer [238,263]. The biocompatibility of chitosan and ability to induce the production of endotoxins has been reported when using scaffold implants in a mammalian model [264]. The early migration of neutrophils into the implantation area, resolved with increasing time following implantation and no other evidence associated with an inflammatory response, such as erythema and oedema in addition to the absence of endotoxin production, was observed [264]. A low incidence of a specific immune reaction determined using a lymphocyte proliferation assay and antibody binding response quantitated using an enzyme-linked immunosorbent assay (ELISA) was observed [264]. The formation of granulation tissue associated with accelerated angiogenesis, a typical healing response, was also reported [264]. The results of this study suggests that chitosan is biocompatible and in agreement with those in which the biocompatibility of chitosan films with different degrees of acetylation was investigated [265,266]. Chitosan oligosaccharides and low molecular weight chitosan exhibited no genotoxicity in lymphocytes [267] and the use of chitosan-coated silver nanoparticles used as an alternative to conventional antibiotics at a concentration of nanoparticles of 3 ppm, did not precipitate a genotoxic effect in mouse macrophage (RAW264.7) cells using a comet assay. However, at concentrations of 20 ppm, a comet result with a considerable tail was observed, suggesting that the genotoxicity was likely concentration-dependent [268].

Synthetic polymers are used extensively in bioengineering and nanoencapsulation applications, as the properties of the materials can be tailored to suit the intended use of the product. Studies to date suggest that PLA and PLGA nanospheres containing bioactive molecules are biocompatible when used in therapeutic applications in vivo, as untoward reactions, either locally or systemically have not been reported [269,270,271,272,273]. The biodegradation of PLA and PLGA nanospheres occurs through homogeneous hydrolytic chain cleavage, where the rate of degradation of the polymer are similar at the external and internal surfaces of the nanospheres [274]. However, the design and development of controlled release nanosphere systems should take cognisance of the potential of the bioactive payload to modulate the hydrate of hydrolysis and tissue response observed [274]. Modified lipid-core nanocapsules (LNC) produced with a negative charge using the synthetic polymer, PCL and polysorbate 80-lecithin inclusion or a positive charge when chitosan was used were tested for haemocompatibility [176] and promising results indicated a good potential for their use as parenterally administered technologies. Furthermore, the negatively and positively charged suspensions when added to blood and no significant haemolysis or platelet aggregation was observed suggesting compatibility, which was independent of the chemical nature of the surface properties of the carrier technology [176]. PEG-coated PLA nanoparticles were tested for an ability to deliver hexadecafluoro zinc phthalocyanine (ZnPcFI_6_) to an EMT-6 mammary mouse tumour model and the polymeric nanoparticles exhibited minimal cytotoxicity to Kupffer cells and reticuloendothelial cells, while accumulation and cytotoxicity in tumour cells was considerable [275]. 

Dendrimers or cascade polymers represent a heterogenic class of compounds consisting of linear or random-coiled polymers, such as PEG, PLA, chitosan and derivative, PLGA, dextrin, hyaluronic acid, PAMAM and PCL [276]. In the systemic circulation, positively charged dendrimers and cationic macromolecules may interact with blood components leading to haemolysis [142], which can induce nephron- and hepatotoxicity [277]. Dendrimer toxicity is dose- and generation-dependent and is related to the presence of a surface charge with cationic dendrimers deemed more toxic than anionic compounds. The inclusion of polyethylene glycol (PEG) or fatty acids may reduce the toxicity of dendrimers, thereby imparting biocompatible characteristics to the complex [278].

The exact mechanism(s) by which nanoparticles precipitate genotoxic effects are largely unknown, however, the composition, size, molecular weight, particle geometry and surface charge may all affect the genotoxic potential of the carriers. Many of these factors are a consequence of the choice and concentration of polymers used when manufacturing the nanocarrier. The genotoxicity of different nanocarriers in a comparative study was primarily affected by the surface charge of the nanocarriers [256], and the extent and degree of genotoxicity was not correlated to the size or molecular weight, as the comparable toxicity observed for nanocarriers vastly different in size which ranged between ~20 nm for PEG to ~600 nm for large “neutral” liposomes [256]. Furthermore, nanocarriers of similar size, molecular weight and shape exhibited substantially different genotoxic effects and particles with a positive surface charge exhibited extensive genotoxicity when the positively charged liposome system was tested [256]. The surface charge of gold nanoparticles (AuNP) plays a critical role in modulating the membrane potential of different malignant and non-malignant cell types and subsequent intracellular events. The authors described a novel mechanism for cell–nanoparticle interactions and AuNP uptake and demonstrated that cellular membrane potential plays a prominent role in the intracellular uptake of AuNP. The agitation of the membrane potential is dependent on the ZP of the nanoparticles, and cationic nanoparticles have the largest impact on membrane depolarization, whereas anionic and neutral nanoparticles appear to have little or no effect. The findings of this study imply that caution must be exercised when using positively charged polymers such as chitosan as they may exhibit genotoxicity. In a study where nanoparticles were synthesized and used at non-cytotoxic concentrations, cationic liposomes, dendrimers, and super magnetic iron oxide nanoparticle (SPIO)-induced genotoxicity that formed significant numbers of micronuclei in cells, whereas anionic and neutral nanocarriers were not genotoxic [256]. The polymeric dendrimers PAMAM and PEI exhibited considerable genotoxicity and require further modification if they are to be used for drug delivery applications [256].

The central nervous system (CNS) is a frequent target for nanoparticulate-assisted drug delivery, however, it is vital and becoming increasingly important to minimize damage to the CNS. In general, it is difficult to penetrate and cross the blood–brain barrier (BBB), as the tightly packed cellular barrier is intended to protect the brain from xenobiotic penetration. However, nanoscale particles manufactured with certain materials of different particle size have overcome this physical barrier or entered the brain through the nerve endings of the olfactory bulb [30,279,280]. 

Neurotoxicity induced by biological, chemical or physical means exert adverse effects on the structure or function of the central and/or peripheral nervous system [281]. Polybutylcyanoacrylate nanoparticles (PBCA-NP) did not induce neuronal death when used in pharmacologically effective concentrations in vivo when coated with surfactants that are known to affect the viability of some cell cultures adversely [282]. The effect of the ZP of anionic and cationic nanoparticles on the integrity of the BBB and permeability of the brain to nanoparticles using in situ rat brain perfusion was investigated, [283] in addition to the evaluation of anionic or neutral nanoparticles and the integrity of the BBB remained intact when exposed to the neutral carriers, whereas high concentrations of anionic and cationic NPs disrupted the BBB and are likely to exhibit immediate toxic effects [283].

Polymeric biomaterials generally exhibit biocompatibility however, caution should be exercised and their use interrogated on a case-by-case basis as the CQAs of the final product may influence different aspects of the biocompatibility of polymeric materials.

### 4.4. Non-Ionic Surfactants

Non-ionic surfactants are capable of forming nanoscale vesicles due to their capacity to self-assemble in aqueous media [284] in addition to exhibiting a good safety profile, biocompatibility and are relatively cheap [285,286]. 

Biomaterials used for the manufacture of drug delivery systems should exhibit minimal toxicity as any technology absorbed into systemic circulation has the potential to precipitate the release of haemoglobin from red blood cells (RBC) which is undesirable and can be fatal, therefore, the determination of the safety of formulations must be established by evaluating potential haemolytic effects of materials [287]. 

Non-ionic surfactants are commonly used for the synthesis of niosomes [33,288,289], SLNs [29,290] and nanocrystals (NCs) [22,291]. More commonly used non-ionic surfactants include Tween^®^ [29,292,293], Span^®^ [33,294,295], D-α-tocopheryl polyethelyene glycol succinate 1000 (TPGS 1000) [27,296,297] and Brij^®^ [298,299,300] and newly synthesized surfactants are being developed and used to improve the CQAs of nanomaterials.

Tween^®^ 80 is a complex mixture of esters and etherates, synthesized separately using oleic acid and ethylene oxide with a two-core matrix of sorbitan and isosorbide. Due to the complexity of the components of Tween^®^ 80, it is difficult to determine the safety of the components and of the exploration of the side effects of the main constituents. An in vitro haemolysis study with LO2 cells and an in vivo acute toxicity assay using zebrafish revealed that nine major components containing a total of 355 chemicals were present and the structures and content were accurately determined [301]. In vitro and in vivo studies revealed that one of the components, polyoxyethylene sorbitan dioleate (PSD) induced the highest rate of haemolysis and greatest cytotoxicity to LO2 cells in addition to resulting in the greatest fatality rate in zebrafish, despite the relatively low amount present in Tween^®^ 80. The results of this exhaustive component-based investigation of Tween^®^ 80 provides a basis for the risk control and improvement of safety and has brought new solutions and ideas for the re-evaluation and application of any complex pharmaceutical excipient.

The biocompatibility of two novel sulphur-based non-ionic surfactants *viz*., DAP-T and DAP-D was investigated using fresh blood for haemolysis testing and in vitro cell toxicity assays [284]. Both surfactants exhibited dose-dependent haemocompatibility with DAP-T resulting in 17.44 ± 1.89% haemolysis at the highest concentration of 1000 µg/mL tested, when compared to 19.65 ± 3.02% haemolysis for the reference material, Tween^®^ 80. Similarly, DAP-D resulted in 14.90 ± 2.27% haemolysis at the highest concentration of 1000 µg/mL, which was <19.65 ± 3.02%, observed with Tween^®^ 80.

The haemolytic and cellular toxicology of a sulfanilamide-based non-ionic surfactant S-SDC in a niosome carrier for hydrophobic drugs has been reported [302] and the S-SDC were found to be haemocompatible with negligible haemoglobin release observed from RBC, even at the highest concentration of 1000 μg/mL tested with 5.78 ± 0.32 and 7.91 ± 0.56% haemolysis occurring with the 500 and 1000 μg/mL sample concentrations, whereas 15.34 ± 0.89 and 19.67 ± 1.29% haemolysis was observed for Tween^®^ 80 at the 500 and 1000 μg/mL concentrations, respectively. The in vitro cytotoxicity of S-SDC investigated in mouse embryonic fibroblast cells NIH/3T3 by MTT assay revealed improved cell viability at the highest concentration when compared to that observed for the negative control viz., Tween^®^ 80 with 93.45 ± 3.78, 86.21 ± 2.41 and 78.84 ± 2.06% cell viability in a concentration range of 30–90 mM, respectively, whereas Tween^®^ 80 resulted in 88.71 ± 2.50, 74.66 ± 3.39 and 62.89 ± 2.65% cell viability in a concentration range of 30–90 mM, respectively.

The biocompatibility of a creatinine-based non-ionic surfactant used in the development of a niosome drug delivery system for clarithromycin was undertaken [303] and 4.40 ± 1.74% haemolysis was observed at the highest concentration of 1000 mg/mL compared to 23.56 ± 2.41% for the reference standard Tween^®^ 80 Studies undertaken using a 3T3 cell line revealed 82.61 ± 3.59 and 80.67 ± 3.81% viability after 24 and 48 h incubation, respectively, at 1000 mg/mL concentration which is better than that for the reference Tween^®^ 80 that exhibited cell viabilities of 65.36 ± 3.98 and 36.61 ± 5.87% after 24 and 48 h, respectively, when used at the same concentration. Similarly, the cell viability was 83.69 ± 2.01 and 80.90 ± 2.11% using HeLa cell lines after 24 and 48 h incubation, respectively, at 1000 mg/mL concentration and the viability of these cells was 56.91 ± 3.71 and 47.56 ± 3.89% using Tween^®^ 80 at 1000 mg/mL. 

The in vivo and in vitro biocompatibility of a novel microemulsion hybridized with bovine serum albumin as a nanocarrier, in which Span^®^ 80 and Tween^®^ 80 were used as surfactants for drug delivery, was reported [304] The haemolytic activity of triacetin microemulsions (T-ME) and triacetin-bovine serum albumin microemulsions (T-BSA-ME) in different concentrations ranged between 6.32 ± 0.02 to 13.00 ± 0.12% and 9.09 ± 0.00 to 15.87 ± 0.00%, respectively. The changes in weight of the mice after 24 h and 7 days was approximately 1.58 ± 3.64% and 5.42 ± 1.78%, respectively. During treatment, none of the mice died, and it was, therefore, concluded using the OCED and Hodge and Sterner scale, that the ME systems were essentially non-toxic.

The haemocompatibility of Span^®^ 80-Tween^®^ 80-based organogels [305] was investigated and the results revealed that haemolysis was <5%, indicating that the organogels containing Span^®^ 80 and Tween^®^ 80 surfactants were haemocompatible. 

Novel fatty acid- and amino acid-based surfactants were synthesized using standard carbodiimide chemistry [306] and biocompatibility evaluated using in vitro haemolytic and cell culture studies and compared to that for commercially available surfactants. The synthetic surfactants exhibited better biocompatibility and similar results were observed in in vivo biocompatibility studies with respect to aspartate transaminase (AST), ALT alanine transaminase (ALT), blood urea nitrogen (BUN), and creatinine serum levels and the histology analysis of the spleen, liver, and kidney in comparison to that for commercially available surfactants Triton X-100 and Tween^®^ 80. The synthetic surfactant also precipitated fewer morphology changes in RBC in vivo.

Sulphanilamide-based long-tail non-ionic surfactants were synthesized and their biocompatibility investigated [307] in vesicles containing ciprofloxacin. The biocompatibility of the synthesized surfactants was assessed using haemolysis and cell cytotoxicity assays and revealed that the synthesized surfactants were haemocompatible, non-toxic and formed spherical vesicles.

In many instances, non-ionic surfactants are used in combination with other biomaterials to produce nanomaterials [296,308,309]. In such instances, it is more complicated to evaluate the combined toxicity of all the components and to establish which of the individual components may cause toxicity. Minimal cytotoxicity to HeLa cells following exposure to lamivudine–zidovudine nano co-crystals stabilized by a combination of TPGS 1000 and sodium dodecyl sulphate (SDS) has also been reported [296].

### 4.5. Ionic Surfactants 

Ionic surfactants are charged amphiphiles that can be classified as anionic, cationic or amphoteric [310] and have been used for many applications as they form solutions with little or no vapour pressure, are biodegradable, are non-flammable, are thermally and chemically stable, have a high solvation ability, and are simple to refine with respect to their physicochemical properties by the alteration of the cation or anion [311]. Despite these advantages, ionic surfactants are not used frequently as non-ionic surfactants as they tend to exhibit toxicity when used orally [312,313]. Cationic surfactants are used less frequently for drug delivery applications, since they exhibit toxicity and are not biodegradable, whereas anionic surfactants are more frequently used due to their negative charge which facilitates targeted delivery [314,315]. 

The use of ionic surfactants provides flexibility as they can be tailored according to the QTPP of products by changing their charge, however, they are known to be haemotoxic and their surface charge interacts with charges on blood cell membranes which alters their toxicological profile [316]. 

Positive or cationic nanoparticles are suitable vehicles for drug and gene delivery, as they readily interact with negatively charged cell membrane surfaces, facilitating the translocation of nanoparticles across the membrane [317]. Negatively charged nanoparticles are thought to repel cell membranes and exhibit poor internalization and are therefore not an appropriate vehicle for drug or gene delivery [318]. 

Despite the toxicity of cationic surfactants, cetyltrimethylammonium bromide (CTAB) and dimethyldioctadecylammonium bromide (DDAB) have been successfully used for the production of drug-loaded nanoparticles [319,320,321,322,323,324] and are promising non-viral vectors for protein delivery by transfection, as they are cost-effective and can be produced on a large scale [325]. The presence of cationic surfaces enhance the bioavailability of the loaded drug, increases the intracellular penetration and the rate of clearance [321,322]. CTAB was used to develop cationic SLNs as a non-viral vector for the delivery of proteins for insulin [326], however, despite the formulation exhibiting 30 day stability and promising results for the delivery of proteins for gene therapy toxicity was a major issue. Cell Proliferation Reagent WST-1 was used to assess the HeLa cell biocompatibility of the cationic formulation for which a reduction in viability, toxicity and increased cytotoxicity was concentration dependant. The in vitro biocompatibility of CTAB and DDAB in five different human cell lines *viz.,* epithelial colorectal adenocarcinoma, liver hepatocellular carcinoma, lung fibroblast, breast adenocarcinoma, and human retinoblastoma was investigated using several routes of administration [327], for which the use of cationic lipids in the SLN formulations influenced the cytotoxicity of the particles significantly, and DDAB SLNs were less cytotoxic following longer exposure times than the CTAB SLNs, for which cytotoxicity occurred even when used in low concentrations. 

Cationic surfactants have been used to impart the charge to liposomes to form cationic liposomes. The toxicity of cationic liposomes is, in part, related to the nature of the cationic surfactant used. The effect of cationic liposomes on human neutrophil activation following the incorporation of cationic surfactants viz., CTAB and soyaethyl morpholinium ethosulfate (SME) in liposomes, induced neutrophil inflammation and toxicity while no neutrophil activation was observed when classic liposomes manufactured using SPC and cholesterol were tested. Neutrophil survival and LDH release revealed that CTAB liposomes exhibited greater cytotoxicity to neutrophils when compared to SME liposomes [209]. 

Anionic surfactants that have been used in microemulsion drug delivery systems include sodium bis-2-ethyl hexyl sulfosuccinate (Aerosol^®^-OT), bile salts/cholates, oleates, sulfosuccinates and Maxemul^®^ 6112 of which Aerosol^®^-OT is most often used due to its versatile application as a surfactant [314]. 

There is a paucity of information relating to the biocompatibility assessment of anionic surfactants and only a few published studies report the comparative effectiveness and toxicity of ionic surfactants [328,329]. The effect of anionic surfactants viz., sodium dodecylbenzene sulfonate, sodium dodecyl sulfate (SDS), non-ionic surfactants viz., polyoxyethylene lauryl ether and Pluronic^®^ F127, cationic surfactants viz., hexadecyl trimethyl ammonium bromide and the Zwitterionic surfactant, lecithin, on the dispersibility and stability of doxorubicin hydrochloride nanodiamond nanoparticles was investigated [330]. The biocompatibility and intracellular delivery of surfactant-modified nanodiamonds and doxorubicin hydrochloride-lecithin dispersed nanodiamonds was assessed for the first time, using the microscopic observation of A549 cells, a radical oxygen assay and the WST assay, and no significant change in the morphology of cells when incubated with 40–160 μg/mL lecithin nanodiamonds was observed. Cell viability for the lecithin nanodiamonds was >75% at an even higher concentration of 320 μg/mL and radical oxygen assay data revealed a limited increase over 8 h, further confirming the biocompatibility of the lecithin nanodiamond with the A549 cells. The cell viability and radical oxygen assay for Pluronic^®^ F127 and polyoxyethylene lauryl ether nanodiamonds (non-ionic) was investigated, however, the biocompatibility of nanodiamonds using cationic and anionic surfactants was not undertaken due to their well-known cytotoxicity [330].

## 5. Conclusions

The inclusion of polymers, lipids, and surfactants for the production of nanocarriers for use in medicinal and non-medicinal application has demonstrated that there is a possibility of producing technologies that exhibit advantages over conventional formulations including improved stability, favourable biodistribution profiles, slower drug release rates, lower immunotoxicity and the potential for targeting specific cells. The use of nanomaterials is growing and thus efficient screening methods for toxicity and biocompatibility are needed to reduce the expense of testing and cost of use. The size, shape, surface chemistry and degree of aggregation of nanomaterials are key factors that influence the toxicity of technologies and in many cases are an integral part of the CQA of the carriers.

These have been investigated and reported for laboratory-scale products and many potentially useful nanomedicines must still transition towards clinical use and application. The success of nanomedicines requires the research, development and characterisation of new formulations to ensure the quality, safety and efficacy of individual nanomedicines which can be attributed to the characteristics of the biomaterials used for their manufacture. Despite an increased understanding of the interaction of nanomaterials with components of the immune system, many questions still remain and require thorough investigation to ensure that a deeper understanding of this phenomenon is gained. 

Herein, we reviewed a wide range of biomaterial literature relating to the materials used for the synthesis of nano-encapsulated drug delivery systems. We reported that, in general, the chemical composition at the surface of nanomaterials influences their interaction with the biological systems, the ability to circulate for long periods in systemic circulation and biocompatibility for use in targeted therapies. The general consensus is that the biomaterial that makes up the surface composition influences the primary toxicity in many cases, whereas internalized biomaterials often contribute to secondary toxicity following the erosion of the outermost surface of the carriers. It is also worth noting that the use of hydrophilic biomaterials, such as PEG, to circumvent the toxicity of nanomaterials that meet all other CQAs may, in their unaltered state, confer additional toxicity or unintended cell delivery to the technologies.

We reported that many but not all aspects involved in the use of nanomaterials are fully understood and, therefore, recommend the development and investigation of appropriate methods for analysis and carefully designed experiments to generate data for a better understanding of the mechanisms of toxicity. In this way, nanomaterial product development would ensure that these technologies can be safely used in biology and medicine. It is our conclusion, that while this review offers a general overview of some uses of the biomaterials used to develop nanomaterials, it is imperative that case-by-case toxicity and biocompatibility analyses are conducted on all the synthesized nanomaterials.

## Figures and Tables

**Table 1:** Common head groups of different phospholipids and their corresponding net charge at neutral pH (Adapted from [197]).

Substituent (R)	Phospholipid	Net Charge at Neutral pH
Hydrogen	Phosphatidic acid (PA)	−1
Ethanolamine	Phosphatidylethanolamine (PE)	0
Choline	Phosphatidylcholine (PC)	0
Serine	Phosphatidylserine (PS)	−1
Glycerol	Phosphatidylglycerol (PG)	−1
Inositol	Phosphatidylinositol (PI)	−1
Phosphatidylglycerol	Cadiolipin (CL)	−2

Key: Anionic at neutral pH, Zwitterion at neutral pH.
